# Role of Artificial Intelligence in the Early Diagnosis of Oral Cancer. A Scoping Review

**DOI:** 10.3390/cancers13184600

**Published:** 2021-09-14

**Authors:** María García-Pola, Eduardo Pons-Fuster, Carlota Suárez-Fernández, Juan Seoane-Romero, Amparo Romero-Méndez, Pia López-Jornet

**Affiliations:** 1 Department of Surgery and Medical-Surgical Specialities, School of Medicine and Health Sciences, University of Oviedo, Avda Julián Clavería 8, 33006 Oviedo, Spain; UO245187@uniovi.es (C.S.-F.); seoanejuan@uniovi.es (J.S.-R.); 2 Departamento de Anatomía Humana y Psicobiología, Biomedical Research Institute (IMIB-Arrixaca), Faculty of Medicine and Odontology, University of Murcia Spain, 30100 Murcia, Spain; eduardo.p.f@um.es; 3 Department of Surgery and Medical-Surgical Specialities, School of Medicine and Dentistry, University of Santiago de Compostela, Entrerríos s/n, 15705 Santiago de Compostela, Spain; mariaamparo.romero@usc.es; 4 Biomedical Research Institute (IMIB-Arrixaca), Faculty of Medicine and Odontology, Hospital Morales Meseguer, Clinica Odontologica Marqués del los Vélez s/n, 30008 Murcia, Spain; majornet@um.es

**Keywords:** oral cancer, artificial intelligence, screening, early diagnosis, machine learning, deep learning

## Abstract

**Simple Summary:**

Oral cancer is characterized by high morbidity and mortality, since the disease is typically in an advanced locoregional stage at the time of diagnosis. The application of artificial intelligence (AI) techniques to oral cancer screening has recently been proposed. This scoping review analyzed the information about different machine learning tools in support of non-invasive diagnostic techniques including telemedicine, medical images, fluorescence images, exfoliative cytology and predictor variables at risk of developing oral cancer. The results suggest that such tools can make a noninvasive contribution to the early diagnosis of oral cancer and we express the gaps of the proposed questions to be improved in new investigations.

**Abstract:**

The early diagnosis of cancer can facilitate subsequent clinical patient management. Artificial intelligence (AI) has been found to be promising for improving the diagnostic process. The aim of the present study is to increase the evidence on the application of AI to the early diagnosis of oral cancer through a scoping review. A search was performed in the PubMed, Web of Science, Embase and Google Scholar databases during the period from January 2000 to December 2020, referring to the early non-invasive diagnosis of oral cancer based on AI applied to screening. Only accessible full-text articles were considered. Thirty-six studies were included on the early detection of oral cancer based on images (photographs (optical imaging and enhancement technology) and cytology) with the application of AI models. These studies were characterized by their heterogeneous nature. Each publication involved a different algorithm with potential training data bias and few comparative data for AI interpretation. Artificial intelligence may play an important role in precisely predicting the development of oral cancer, though several methodological issues need to be addressed in parallel to the advances in AI techniques, in order to allow large-scale transfer of the latter to population-based detection protocols.

## 1. Introduction

Oral cancer is characterized by one of the poorest cancer survival rates worldwide—a situation has not improved despite the recent therapeutic advances made. According to GLOBOCAN, lip and oral cancer had an incidence of newly diagnosed cases in the year 2020 of 377,713 cases; 264,211 male and 113,502 female, with mortality 177,757—125,022 male and 52,735 female [1]. Many cases of oral and oropharyngeal cancer are detected in advanced stages of the disease, resulting in needless morbidity and mortality [2,3]. The key factor in this regard is detection of the lesions as soon as possible, while they are still in an early stage, in order to improve the chances for successful treatment. Cancers that are detected late or which prove less accessible are associated with poorer survival, greater treatment-related problems, and increased medical care costs [4,5,6,7].

Improved knowledge of the disease and of its risk factors and symptoms would have a positive influence upon the diagnosis, facilitating the identification of potential symptoms of malignancy which otherwise might be undetected or not be adequately evaluated [2,3,4,5,6,7,8]. Due control in turn is required for established risk factors such as smoking and alcohol abuse, together with the detection of human papillomavirus (HPV) in relation to oropharyngeal cancers [5].

OPMD have been defined as “any oral mucosal abnormality that is associated with a statistically increased risk of developing oral cancer”. The following pathologies are considered OPMD: oral leukoplakia, proliferative verrucous leuokolakia, erythroplakia, oral submucous fibrosis, oral lichen planus, actinic keratosis, palatal lesions in reverse smokers, oral lupus erythematosus, dyskeratosis congenital, epidermolysis bullosa, oral lichenoid lesion and oral chronic graft vs. host disease [9].

It is important to identify lesions that may undergo malignant transformation. In this regard, visual screening of the oral cavity has been widely recognized as a viable, safe and precise strategy for detecting such lesions with a view to reducing oral cancer mortality [5,6,7]. At present, the diagnosis is based on a thorough clinical exploration—the latter forming part of any routine medical consultation, affording high discriminating capacity and taking little time to complete in the clinic [2,3,4,5]. Several recent studies have evaluated the use of autofluorescence in the context of population screening interventions, and recommend it as an adjunct to conventional oral examination for the assessment of oral potentially malignant disorders (OPMDs), with oral biopsy remaining the diagnostic gold standard in all cases [10,11,12,13,14].

A late diagnosis of oral cancer occurs as a result of a complex interaction of multiple interrelated factors. In this respect, different authors have defined four problems that should be targeted for corrective actions: (a) late detection of the symptoms; (b) scant knowledge of oral cancer; (c) delays in seeking medical care; and (d) the focusing of interventions upon concrete risk groups [7,8].

The field of healthcare is experiencing unprecedented changes thanks to the technological advances of recent years. The introduction of digital medicine is expected to modify the practices of healthcare professionals as a result of increased interaction with the new information and communication technologies [15,16,17]. Thanks to artificial intelligence (AI), we will have tools allowing us in a matter of seconds to analyze and interpret a great volume of data, helping us in the decision-making process. Innovations in digital technologies offer clear benefits for healthcare professionals, healthcare systems, and patients [18].

Artificial intelligence is beginning to have a considerable impact, improving diagnostic precision in certain medical fields, and may be of great help in all aspects related to the oncological workflow–from screening to patient treatment [18,19,20].

Artificial intelligence may be defined as the capacity of software to imitate the cognitive abilities of humans. Machine learning (ML) is a part of AI that is centered on the use of algorithms to solve different problems, including data classification or regression, and is an emerging area of interest for investigators seeking to transform large bodies of data into knowledge that may be of use in clinical decision making. In ML, the algorithms require no prior explicit programming to operate. Machine learning can be classified according to the type of learning as follows [18]:

1. Supervised learning: the training process in this case is based on labeled data using a known external standard known as the “ground truth”.
2. Unsupervised learning: the algorithm analyzes unlabeled data to identify hidden structures. In this case, the algorithm itself seeks to detect patterns in the data for learning, since the system lacks prior labeled data or expectable results.
3. Reinforcement learning: in this case, the software actions receive positive and/or negative reinforcement within a dynamic environment.

In medicine, supervised learning is the most widely used form of ML. Unsupervised learning generally requires a large body of data, and the results may be complex to interpret. Reinforcement learning requires a trial-and-error process that is difficult to implement in the health sciences; at present it is mainly applied in robotics, telecommunications and game theory [18,19,20].

The use of ML has grown in recent years thanks to technological advances that have allowed increased patient data digitalization through electronic case histories and image files, as in the fields of Radiology and Pathology. A recent tendency has been the growing use of radiomics—a computational tool of help in establishing the diagnosis, and which fundamentally involves imaging data conversion to detect differential features not apparent to the human eye. Such new imaging characteristics may be of diagnostic, prognostic, and therapeutic usefulness [15,16,17,18,19,20,21].

Deep learning (DL) is the most recent evolution of ML, and is more appropriately described as a sub-discipline of ML. Its functioning is more complex, and it is able to afford decision-making capacity and process extremely large data sets [18,19].

A body of ML algorithms of particular interest in the recent literature is referred to neural networks (NNs). These are complex models composed of nodes (called neurons) that model deep networks characterized by several layers. The use of NNs with this architecture is commonly known as deep learning. This technology allows high-level abstraction of the input data, with great performance in different tasks ranging from the analysis of images to personalized drug design [18].

AI has led to significant advances and developments in oncology [17,18,19]. Different narrative reviews have been published in relation to their usefulness for facilitating the early diagnosis of OPMD and oral cancer [22], and for the support they provide for the same purpose, as well as radiological, endoscopic, spectrometric or histological images [23,24,25]. In this regard, the present study was carried out to provide a scoping review of the application of AI to the early diagnosis of oral cancer using non-invasive techniques as well as the proposal for future investigations.

## 2. Materials and Methods

### 2.1. Protocol and Registration

This scoping review was registered as a protocol with the PROSPERO (International Prospective Register of Systematic Reviews) platform (registration number: CRD42020218675). No ethics committee approval was required for the present systematic review. 

The question proposed was as follows: What are the applications and performance of artificial intelligence in the early diagnosis of oral cancer? 

### 2.2. Search Strategy

The review was based on the PRISMA ScR (Preferred Reporting Items for Systematic reviews and Meta-Analyses extension for Scoping Reviews) statement [26]. The literature search was performed in the PubMed, Web of Science, Embase and Google Scholar databases. The following terms were combined to identify relevant publications: “oral cancer”, “oral precancer”, “oral potentially malignant disorder”, “oral leukoplakia”, “artificial intelligence”, “deep learning”, “machine learning”, “convolutional neural network”, “artificial neural network”, “diagnosis”, “screening”, “telemedicine”, and “mobile”. All the identified studies were evaluated by two blinded reviewers (PLJ, EPFL) on an independent basis. In case of disagreement, a third reviewer (MGP) was consulted. Reference lists were also screened for additional studies.

### 2.3. Eligibility Criteria. Inclusion Criteria

We included papers focused on the use of AI in the early noninvasive diagnosis of oral cancer, in which the measurement of effectiveness was included in the results, covering the period from January 2000 to December 2020. There were no language or study design limitations.

### 2.4. Exclusion Criteria

We excluded articles related to AI but based on radiological imaging (computed axial tomography (CAT), magnetic resonance imaging (MRI)), biomarkers, metastasis, recurrences and survival, or the planning of treatment; articles unrelated to AI; articles not published; and articles based on animal experimentation.

### 2.5. Data Items

Data were extracted from original articles using a set of predetermined parameters. The following data were compiled: year of publication, country, research objective concerning the diagnosis of OPMDs or oral cancer, sample size, AI tool used for oral cancer and precancer diagnosis and classification methods, as well as the quantitative results obtained from their evaluation.

### 2.6. Critical Analysis and Evidence Synthesis

The analysis was divided into sections addressing oral cancer screening, optical imaging and enhancement technology, and oral cytology. The review focused on summarizing the evidence on the application of AI for the detection of OPMDs and the early diagnosis of oral cancer. The formulation of the questions performed for each oral cancer diagnostic tool selected in this review, were the following:

Q1. In relation to telemedicine (teledentistry or telehealth)
Q1a. Is there agreement in the diagnosis of oral lesions between the practitioner and experts in Oral Medicine or Oral Cancer?
Q1b. Would the images received by mobile (telemedicine), and classified through the neural network, corroborate the diagnosis of OPMD and oral cancer?
Q2. Would the classification of photographic images submitted to AI allow the discrimination of OPMD and oral cancer?
Q3. Does the application of light-based detection on the lesion improve the AI classification of lesions for decision-making in the diagnosis of OPMD and oral cancer?
Q4. Does exfoliative cytology offer information for the screening of patients at risk of oral cancer?
Q5. Do the demographic variables of the patients, the toxic habits, and the clinical parameters, introduced in the IA classification models provide predictive values for oral cancer?

## 3. Results

### 3.1. Selection of Resources/Search Results

**Figure 1:**
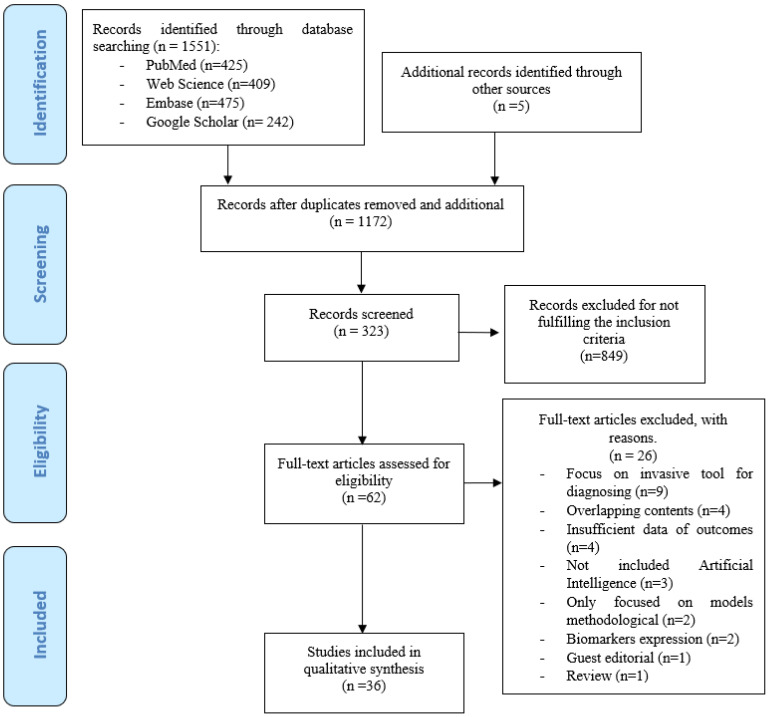
Flow diagram of the scoping review.

The first step resulted in the preliminary identification of 1551 articles. A total of 384 publications were found to be duplicates and were discarded, thus leaving 1167 articles of which 1110 were excluded after evaluation of the title and abstract. In the second step, and after full-text reading of the 62 selected articles, a total of 36 were included in the review [27,28,29,30,31,32,33,34,35,36,37,38,39,40,41,42,43,44,45,46,47,48,49,50,51,52,53,54,55,56,57,58,59,60,61,62], with a description of the relevant findings (Figure 1).

### 3.2. Description of Studies

Only five of the selected articles were published before 2015 [33,38,44,58,59]. The field work of 12 studies was carried out in India [29,30,34,39,44,49,51,52,54,55,56,57], while five studies were carried out in Malaysia [37,42,47,48,61] and in China [28,35,43,59,60], four in the United States [36,38,45,46], two in The Netherlands [33,58] and Poland [40,41], and one in Taiwan [32], Saudi Arabia [50], Morocco [31], Germany [27], Greece [53] and Sweden [62].

The study subjects and aims were classified as follows: (1) identification of the most appropriate biopsy site [28]; (2) selection of patients through clinical screening and the referral of suspicious cases to a specialist in oral medicine or oral cancer [30,37,52,57,61]; (3) screening in smokers [34,49]; (4) oral cancer screening through smart telecytology [54]; (5) the diagnosis of OPMDs such as solar cheilosis [53], oral lichen planus [41,46], leukoplakia [29,40,58], with a prediction of their course [43]; (6) aids to differential diagnosis by classifying the lesions as benign or precancerous [50]; normal mucosa or oral cancer [27,32,35,36,39,42,62]; or as different benign, premalignant and malignant lesions [31,33,39,46,59]; (7) classification of oral cancer [56]; and (8) development of oral cancer risk predictive models [42,47,48,51,55,60].

The most frequent evaluative metrics were: concordance [30]; precision [31], sensitivity, specificity [29,40,41,43,44,53,58,60]; concordance, sensitivity, specificity [37]; accuracy [47,56]; accuracy—area under the curve (AUC) [45,46,55], accuracy, sensitivity, specificity [28,34,36,38,39,42,49,52,54]; receiver operating characteristics curve (ROC-AUC) [33]; accuracy, sensitivity (recall), specificity, F-measure, ROC-AUC, precision [51]; precision, recall and F1-score [61]; sensitivity, specificity and ROC-AUC [45,57]; sensitivity, specificity and IOU (intersection over union evaluating accuracy of the ROI (region of interest)) [32]; accuracy, sensitivity, specificity and ROC-AUC [27,35,48,50]; accuracy, precision, recall and F-score [62]; sensitivity, specificity and positive predictive value [59]. The definitions of the terms employed are provided in Table 1.

The number of risk factors or attributes used to construct the predictive models employed as criteria for the referral of suspicious cases ranged from 5 [63] to 25 [47]. The most frequent were: demographic data (age and gender) [43,47,48,51,55,61]; a cut-off age of under 40 years [30,42]; toxic habits (smoking, alcohol and tobacco chewing) [30,42,43,47,48,55,61]; and clinical parameters (location [43], three-week red or white lesions [30], and ulcerations with pain for over 14 days [47]). Other considered factors were ethnic group [42,47], limited oral opening [30], neck adenopathies [30,51], comorbidity [51], and the diagnostic technique employed, among others [60]. 

#### 3.2.1. Mobile Phone Technologies

Mobile phone technologies were used in six studies as instruments for the screening and diagnosis of suspicious oral lesions (Table 2) [30,37,52,54,57,61]. Birur et al. [30] established interactive remote consultation between frontline health care workers (FHWs) and primary care dental practitioners and specialists in oral cancer. This strategy resulted in concordance in the imaging diagnosis of suspicious lesions in 45.1% of the FHWs, and concordance was confirmed in 100% of the cases with the primary care dental practitioners [30]. Such concordance was maintained in the study published by Haron et al. [37], with a specificity of 100% between dentists and specialists in oral medicine in relation to the analyzed parameters (presence of lesion, category of lesion and referral decision) [37].

Song et al. [52] and Uthoff et al. [57] equipped smartphones with an external light-emitting diode (LED) system and a combined autofluorescence imaging (AFI) and white light imaging (WLI) application. Using this strategy with transfer learning (VGG-CNN-M), the authors achieved superior validation of the images for distinguishing between suspicious lesions (malignant and premalignant) and non-suspicious lesions (normal and variants of normal) compared with the separate use of the applications [52]. With this same methodology, on comparing the interpretation of the remote specialist and different transfer learning CNN (convolutional neural network) strategies, greater sensitivity was recorded with the remote specialist (92% vs. 85%, respectively), though specificity proved greater with the CNN strategy (85% vs. 88%) [57]. However, in a recent study [61], although classification and detection with ResNet and Faster R-CNN yielded high specificity (93.8%) in determining whether the lesion in the image requires referral to specialized care, evaluation of the discrimination between low risk OPMDs and high-risk lesions or cancer found the specificity to be lower (43.9% vs. 56.0%).

#### 3.2.2. Medical Imaging Techniques

The analysis of medical images for the early detection of oral cancer was performed in nine studies (Table 3) [31,32,35,39,40,41,50,53,56]. The use of Speed Up Robust Features (SURF) in Support Vector Machine (SVM) allows the differentiation between normal and pathological mucosa with a precision of 82% [31]. Shamin et al. [50] found that in classifying benign and precancerous lesions of the tongue, pre-processing with the VGG19 model afforded greater accuracy (98%). Spyrodonos et al. [53], using the Relevance Vector Machine, recorded a specificity of 96% for the identification of solar cheilosis vs. non-solar cheilosis. 

Application of the Probabilistic Neural Network allowed the differentiation between oral lichen planus, leukoplakia and normal tissue, with a specificity of 81%, 74% and 88%, respectively [41]. The specificity with respect to leukoplakia improved to 97% by applying textural features such as wavelet energy for segmentation of the constituent layers [40].

The distinction between normal tissue and oral squamous cell carcinoma (OSCC) using the CNN strategy yielded an accuracy of 92.3% [35], and of 94.5% with partitioned deep CNN [39]. Likewise, following analysis and the use of textural filters, distinction between normal tissue and OSCC was achieved with a specificity of 0.9475 in identifying ROI [32], with further improvement being obtained by selecting 11 gray-level co-occurrence matrixes (GLCMs) (accuracy 97.9%) [56].

#### 3.2.3. Fluorescence Imaging

In addition to the two articles mentioned above [52,57], another nine studies incorporated luminescence to AI as a noninvasive method for the diagnosis of oral precancer and cancer (Table 4) [27,28,33,36,38,44,58,59,60]. For the diagnosis of OSCC, Aubreville et al. [27] used confocal laser endomicroscopy, which affords high magnification of the mucosal surface, yielding a specificity of 90% and an accuracy of 88.3%. Majunder et al. in turn used N_2_ laser with a specificity of over 92% [44].

Illumination based on fluorescence emission with the VELscope enhanced oral assessment system was used to identify the most appropriate biopsy site in dysplastic areas (accuracy 83%) [28] and to determine the risk factors for OPMD transformation [60]. This latter study made use of a customized model (model P) considering different factors that could concur in progression towards oral cancer—the most closely related being use of the VELscope and blue toluidine staining, and patient age [60].

Xenon white-light illumination was used in five studies [33,36,38,58,59]. It has been suggested to be useful in diagnosing leukoplakia [58] and for facilitating identification and differentiation between oral submucosal fibrosis (OSF) [59], other OPMDs [38] and oral cancer, as well as between healthy tissue and carcinoma [27], and for intraoperative cancer detection [36].

Wang et al. classified premalignant and malignant lesions vs. benign lesions, with a sensitivity of 81% and a specificity of 96% [59]. The results showed improved identification of OSF (accuracy 97%) [59] in comparison with differentiation between homogeneous and non-homogeneous leukoplakia (sensitivity 73% vs. 64%, and specificity 82% vs. 94%) [58], while de Veld et al. were unable to discriminate between benign and premalignant lesions [33].

#### 3.2.4. Exfoliative Cytology

Cytological diagnosis was used in nine articles, based on exfoliative liquid [29,49], scraped [34] and brush biopsies (Table 5) [43,45,46,52,62]. Banerjee et al. [29], using linear SVM, classified oral leukoplakia and OSCC cells with a sensitivity and specificity of 100% when only using the cellular descriptors, vs. a sensitivity of 89.9% using the nuclear descriptors. However, Sunny et al. [54], using smart cytology with remote diagnosis for distinguishing between OSCC and HGD (high grade dysplasia) vs. LGD (low grade dysplasia), recorded an accuracy of 60% with manual assessment by the professional, vs. 90% using an artificial neural network (ANN)-based risk stratification model. The authors underscored that the number of images needed to diagnose OSCC may be less than 20, while over 100 images might prove necessary in the case of dysplasia [54].

Liu et al. [43], using the peak detection–random forest model, were able to predict the malignant transformation of leukoplakia with a sensitivity of 100% and a specificity of 99.2%, thereby improving upon the previously used model with SVM.

Cellular classification with SVM allowed distinction between the cells of healthy smokers and those of individuals with oral leukoplakia and OSCC, recording an accuracy of 85.71% [34] and a positive correlation coefficient of 0.86 between smoking duration among patients with OPMD and early cancer risk [49].

McRae et al. [39] applied logistic regression analysis based on CellProfiler software, with an AUC of between 0.81 and 0.97, the former value corresponding to the dichotomic model of benign lesion vs. dysplasia, and the latter to no lesion vs. malignant lesion. The authors also found nuclear F-actin staining to be associated with early disease (lower proportion in benign lesions), with oral lichen planus being associated with lesser staining. Late disease models proved more accurate (AUC 0.88–0.97) than early disease models (AUC 0.77–0.87) [46].

Wieslander et al. [62], on comparing two different network architectures for discriminating between normal mucosa and cancer, recorded an accuracy of 80.66% and 78.34% with VGG and ResNet, respectively, observing that VGG classifies more tumor cells and more healthy cells as being malignant than ResNet.

#### 3.2.5. Predictor Variables of Datasets

Five studies constructed algorithm patterns involving attributes or variables compiled from databases of oral cancer patients to select cancer risk predictors (Table 6) [42,47,48,51,55]. The accuracy values differed depending mainly on the number of attributes and the type of algorithm selected. Thus, in the comparative study published by Tetarbe et al., the best algorithms for detecting oral cancer were the REPTree and the J48Tree (78.7% vs. 77.6%) [55], while Mohd et al. recorded the best performance for the Multilayer Perceptron (MLP) (94.7%) [47], with 18 and 14 attributes, respectively. In other studies, the distinction between benign and malignant lesions based on Fuzzy regression or logistic regression analysis yielded accuracy values of between 78.9% (8 attributes) [42] and 99.3% (12 attributes) using Probabilistic NN and General Regression NN [51].

On contrasting the results obtained based on classification by the oral cancer clinician and the fuzzy neural network and fuzzy regression analysis predictive models, no statistically significant differences were recorded in the analysis of one or two risk factors, though significant differences were observed between the clinician and the fuzzy models in relation to three and four factors [48].

### 3.3. Artificial Intelligence (AI) Methods Used in Selected Studies

Most of the studies combined different supervised learning methods, with a lesser use of unsupervised learning methods—the latter being the most commonly used strategy for the measurement of cellular and nuclear size indices in cytological studies.

On comparing supervised with unsupervised learning methods, principal component analysis (PCA) with ANN, Veld et al. [33], separating the red/green intensity ratio, found ANN to yield a slightly greater ROC-AUC of 0.90–0.97 in differentiating cancer from healthy tissue, though the AUCs in distinguishing between premalignant lesions or other benign lesions were very small.

Among the studies that used predictive variables for early diagnosis based on the information contained in the databases, only one article used k-nearest neighbor (KNN) [47]. Mohd et al. [47] adopted the synthetic minority oversampling technique (SMOTE) algorithm and found SVM to outperform other machine learning algorithms such as Bayes (NB), KNN and multilayer perceptron (MLP). To achieve greater accuracy, the authors recommend reducing the number of attributes or patterns included in the algorithm–the best outcomes being observed with seven attributes [47].

Textural analysis of the images showed classification based on patch-probability fusion CNN to be better than textural classification using Random Forest or SVM with local binary patterns (LBPs) and gray-level co-occurrence matrixes (GLCMs) [27].

With regard to the textural filters used to improve the classifications, and apart from GLCMs and gray-level run-length (GLRL) for the classification of oral cancer [56] and discrimination between cancer and normal mucosa, Chan et al. [32] found that on applying the texture-map-based branch-collaborative network, the Gabor filter afforded greater information for the detection of cancer and greater sensitivity and specificity than analysis based on the wavelet transform. Awais et al. [28], using the KNN (k-nearest neighbors) classification, found the highest accuracy (83 ± 5%) to be obtained with a combined pattern of variance, correlation, inverse different moment, sum average, sum variance, sum entropy, entropy and difference entropy. It had previously been reported that in application to photographic images, specificity for leukoplakia improved with wavelet energy analysis [40,41].

Shamim et al. [50] found pre-processing with VGG19 to afford greater accuracy, sensitivity and specificity in distinguishing between benign and precancerous lesions when compared with AlexNet, GoogLeNet, ResNet50, Inceptionv3 and Squeeze Net. However, ResNet yielded better results in distinguishing between different tongue lesions. Improved performance was also observed when this strategy was used in exfoliative cytology [62].

In analyzing behavior with xenon light, Halicek et al. [36] used an image implementing system with Tensor Flow, prior to classification, recording the best accuracy in differentiating between health tissue and cancer with the CNN classification (96.4%), followed by SVM, KNN, LR, DTC and LDA (67.4%).

Rosma et al. [48], on comparing the prediction of oral cancer, found Fuzzy Neural Network models to be more specific, and Fuzzy regression prediction analysis yielded greater accuracy and sensitivity, but lesser specificity, than interpretation by clinicians. Sharma et al. [51], using probabilistic neural network (PNN) and general regression network (GRNN) programs, documented higher percentage performance in differentiating between benign and malignant lesions according to the diagnostic attributes used, and in validation compared with linear regression, decision tree forest, tree boost, MLP and CCNN. In contrast, Tetarbe et al. reported the best accuracy performance with random tree [55].

Another contribution has been the observation that the use of a low-resolution camera in recording the images with a mobile phone results in more false negative results, thus justifying the use of mobile phone cameras with a resolution of 720 × 1280 or 1080 × 1920 pixels [37].

## 4. Discussion

The present review analyzed 36 studies using different machine learning techniques as an adjunct to the noninvasive diagnosis of oral precancer and cancer. The methodological heterogeneity of the studies, with diverse definitions, sample selections and sizes, different CNN classification protocols, and differences in assessing their validity, precluded the conduction of a meta-analysis.

Most of the studies were published after 2014 and they were concentrated in Asia (28/36 articles), a region characterized by the highest lip and oral cancer incidences in the world [63]. The tools derived from deep learning constitute a noninvasive adjunct to the early diagnosis of oral precancer and cancer, not only for dentists, but also for primary healthcare practitioners. On the other hand, the use of databases to identify those attributes most closely related to oral cancer could represent an advance in the selection of individuals for screening purposes.

Teledentistry based on the use of mobile phones was addressed by six of the studies [30,37,52,54,57,61], affording a connection between primary health care professionals or dentists and specialists in oral medicine or oral cancer. Recently, Ilhan et al. highlighted the role that AI could play in reducing in oral cancer diagnosis delay, especially telemedicine in low-resource settings. [22]. To the question raised in this review about the concordance between oral disease explorers and specialists at the cancer center, to recognize OPMD lesions, the answer is that this agreement exists in 100% when the explorers are dentists but has a predictive value of 45% when they are frontline health workers [30]. The sensitivity is lower among dentists than among experts in oral cancer in specifying the presence of the lesion, the category of the lesion, or the decision to refer patients, at 70% and 81%, respectively [37]. The incorporation of fluorescence techniques or the use of cameras that improve the quality of the images and facilitate their subsequent processing constitutes an improvement in the design of databases linked to mobile phones. Using this type of light, and in relation to the question of the classification of the images captured by mobile and classified by a specialist oral oncologist and subsequent classification with the VSG-CNN-M model, this is comparatively better than the VGG-CNN -S and VSG-CNN, 16 [52], achieving a sensitivity of 85% [52,57]. Sensitivity is lower when annotations of demographic and risk factors are incorporated into the classification regarding the need to refer both low-risk OPMD and high-risk OPMD or cancer (43% and 56%) [61]. These resources could be a great advantage in first screening in those settings where not only is the incidence of oral cancer high, but the available healthcare resources are limited, reducing unnecessary referrals [64] and shortening distances between patients who need specialized diagnoses and the specialist [65]. Furthermore, the use of artificial neural networks (ANNs) has also been described as a measure of support for the remote cytological diagnosis of malignant lesions and high-grade OPMDs [64], contributing to lessening the difficulties posed by photographic images [61].

Different studies have used clinical photographs to demonstrate that lesions suspected to correspond to OSCC can be easily and automatically differentiated by applying an algorithm [31,32,35,40,41,50,53,56]. Thus, practitioners have a practical, noninvasive and profitable tool open to non-specialists for the detection of OSCC, and thus for improving the prognosis of oral cancer. In the field of dermatology, AI is helping with the diagnosis of precancerous lesions, and carcinomas such as basal cell carcinoma and melanoma [66], obtaining through methods to extract the texture features an accuracy for the diagnosis of melanomas of 98.35% [67], and an AUC of 0.81 [68]. However, the variability of the photographic images poses a problem for the identification of oral cancer or OPMDs, and this scenario is much more complicated than the classification of skin lesions, since the assessment of lesions within the oral cavity is often conditioned by interference from teeth, the oral mucosa, tongue, lips and palate.

With respect to the question of the discrimination by classifying OPMD and oral cancer images, photographic images offer a high distinction between OSCC and benign lesions (accuracy 94%), and also between OSCC and normal tissue, with internal validation of 88.7% [35]. Regarding OPMD, the specificity is higher for solar cheilosis (96%) [53] than for oral lichen planus (81%) [41]. It should be noted that Jurczyszyn et al. achieved better results for the diagnosis of leukoplakia applying a greater number of textures features, both in sensitivity (57% vs. 100%) and in specificity (74% vs. 97%). [40,41]. Despite recent advances in deep learning techniques for medical imaging interpretation purposes, large volumes of data are needed in order to secure the required diagnostic performance. In contrast to computed tomography (CT) or magnetic resonance imaging (MRI), oral photographs are not mandatory before treatment [10,20]. In practice, this means that it is extremely difficult to compile large amounts of photographs, but it would allow comparative studies. The indicators in the improvement of the results of the analyzed studies are based on the combination of deep CNN and texture filters such as Gabor, sunlight matrix, co-occurrence matrix, or different grey level matrixes.

Regarding the question of the use of luminescence (e.g., xenon light) to improve the registries, more favorable results were yielded in the dichotomous discrimination between normal and pathological images, or between normal tissue and cancer, than in establishing differences between benign and premalignant lesions [33], where accuracy has been found to be poorer. This method is also useful for predicting the progression of precancerous lesions towards cancer [54], for diagnosing oral submucosal fibrosis (OSF) [32] and for leukoplakia [58].

A recent systematic review showed that the vascular changes suffered in the chorion and submucosa capillary loop microvascular architecture, observed through narrow-band imaging (NBI), provide greater reliably for the diagnosis of premalignant oral lesions and oral cancer than using white-light imaging [69]. Segmentation of NBI videos by AI has been used for the diagnosis of oropharyngeal cancer [70,71] and for oral precancer and cancer [72]. Paderno et al., in a publication this year, stated that by applying the fully convoluted neural network for the segmentation of video-endoscopic images, values of 0.6559 could be obtained for the dice similarity coefficient [72], so despite not having been included in the present study, the NBI also seems a promising tool for the diagnosis of oral cancer.

However, in answer to this third question, it must be taken into account that while fluorescence may be an adjunct or complement to oral examination in the diagnosis of oral precancer and cancer [73], it cannot be postulated as a substitute for biopsy [74]. This affirmation was ratified in the last Cochrane review, in which it was stated that none of the complementary tests, such as vital staining, oral cytology, light-based detection, and oral spectroscopy, replace biopsy for the diagnosis of oral cancer [75].

Another question analyzed was focused on whether exfoliative cytology provides information for the screening of patients at risk of oral cancer. Support vector machine (SVM)-based classification can be used in decision making as a noninvasive technique using exfoliative cytology or LBEC (liquid-based exfoliative cytology) samples to establish oral leukoplakia and OSCC with high sensitivity and specificity. Exfoliative cytology also affords relevant information for early diagnosis in smokers [34], and for monitoring lesion progression towards malignancy [49]. Therefore, this must also be considered for a first screening in smokers.

The last question that has been raised is focused on the attributes or variables that could be considered to carry out the screening of patients at risk of developing oral cancer. This aspect has been approached from the point of view of the number of attributes and from the qualitative variable. It has been highlighted that in order to generate better accuracy, it is important to reduce the number of variables of the algorithm [47]. Regarding the type of variable, Rosma et al. described for drinkers an AUC of 0.724 determined by clinicians and 0.713 in the fuzzy classification, and when drinking and chewing tobacco are associated it is 0.78 and 0.76, respectively [48]. Mohd et al. presented an accuracy of 94.76% in the analysis of 14 attributes, including, besides other histopathological parameters, the clinical ones of gender, ethnicity, site, size, painful and painless ulceration > 14 days [47]. Due to the reduced number of published articles, new studies must be carried out to assess demographic parameters and toxic habits of great relevance for the selection of patients to be screened.

The confusion matrix delimits the evaluation of a supervised deep learning algorithm. Most of the studies in the present review based their evaluation on sensitivity, specificity and accuracy, though other metrics are available that afforded validity to the CNN process. It is advisable for future studies to take into account the TRIPOD (Transparent Reporting of a Multivariable Prediction Model for Individual Prognosis or Diagnosis) criteria [76], with standardized clinical trial protocols for interventions involving artificial intelligence as referenced in the SPIRIT-AI (Standard Protocol Items: Recommendations for Interventional Trials—Artificial Intelligence) guide, in order to adequately interpret methodologically homogeneous results [77].

The analyzed articles have several limitations: (1) six studies involved small sample sizes (fewer than 30 patients) [27,36,37,56,58,62], in the context of deep learning; (2) in the study carried out by Shamim et al. [50], the images were retrieved from the Internet, while Fu et al. [35] based external validation on images from six representative journals in the field of oral and maxillofacial surgery and dentistry; (3) images of the side contralateral to the side of the lesion were regarded as representing healthy tissue [27], or healthy tissue was considered to correspond to individuals who in principle were healthy but had toxic habits (e.g., the chewing of areca nuts), and thus could already present mucosal alterations; (4) not all the studies corroborated the clinical diagnosis with the biopsy findings [44,46], and (5) since it is an emerging topic, there is a limitation due to the time limit of the search for publications.

We must also point out as knowledge gaps that the available evidence is not enough to validate any of the diagnostic tools analyzed or deep learning in the diagnosis of certain precancerous lesions. Specific data were provided in five papers on the analysis of oral leukoplakia [29,40,41,43,58], one on actinic cheilosis [53], one on oral lichen planus [41], and another on oral submucous fibrosis [32].

## 5. Conclusions

Artificial intelligence will greatly remodel studies on the early detection of oral cancer, and consequently will improve clinical practice in general. Artificial intelligence offers excellent opportunities for the automation of tasks through the detection of complex patterns. In this respect, research is crucial to facilitate the interdisciplinary incorporation of such techniques, and improvements in this field may open the door to further studies in the future.

## Figures and Tables

**Table 1:** Common terminology used in artificial intelligence.

Term	Interpretation
Artificial intelligence (AI)	A field of science and engineering concerned to develop machines that can learn through data so that they can solve the problems.
Machine learning (ML)	A subfield of AI in which algorithms are trained to perform tasks by learning patterns from data so they can resolve issues without human input.
Deep learning (DL)	A subset of machine learning. The purpose of DL is to construct a neural network that automatically identifies patterns to improve feature detection, collecting features from the abstracted layer of filters.
Neural Network	A set of algorithms of solutions to a problem that compute signals via artificial neurons, to create neural networks that function like the human brain
Probabilistic systems	Incorporate rates of diseases or problems in a population and the likelihood of various clinical findings in order to calculate the most likely explanation for a particular clinical case
Supervised learning	Based on labeled data using a known external standard called as the “ground truth”. A model is built by learning common features from a non-labeled set of training data
Unsupervised learning	The algorithm itself seeks to detect patterns in the data for learning, since the system lacks prior labeled data or expectable results. Model is built by learning common features from a non-labeled set of training data.
True positive	An abnormal lesion is categorized correctly as abnormal.
True negative	A normal is categorized correctly as normal
False positive	A normal is categorized wrongly as abnormal
False negative	An abnormal is categorized wrongly as normal.
Accuracy	The proportion of correctly predicted results among all samples, the proportional precision in a classification system. Test accuracy 0.90, the model correctly classified 90%.
Sensitivity (recall)	The ratio of true positives to total positive predictions or the proportion of the true cases that are identified by the model. Percentage predicted positive among all truly positive
Specificity	The ratio of true negatives to total positive prediction Percentage predicted negative among all truly negative
Precision (positive predictive value)	The proportion of cases selected by the model that has the true value. The proportion of the patients with the disease, who are correctly predicted to have the disease. The number of true positives divided by the number that was predicted as positive
F1 Score	The harmonic mean of the precision and recall
Receiver operating characteristics (ROC)	A curve for a model and is used for estimating the prediction ability of a model.
Training	Used for generating or created a model
Validation	Used to estimate the model effectivity or prediction error

**Table 2:** Mobile technologies. ^1^ Country of field work. AFI: Autofluorescence imaging. CNN: convolutional neural network. FHW: frontline health care workers. OPM: oral potentially malignant. OPMD: oral potentially malignant desorders. WLI: white light imaging. ^2^ Suspicious: leukoplakia, lichen planus, oral submucous fibrosis. ^3^ Suspicious: OSCC, lichen planus, homogeneous leukoplakia, speckled leukoplakia, tobacco pouch keratosis, verruccous leukoplakia, oral submucous fibrosis.

Authors, Year, Country, ^1^ Reference	Aim	Method. Classifier	Sample	Outcomes: Diagnostic Performance (%)
Birur et al., 2015, India [30]	To determine the effectiveness of a mobile phone–based for a surveillance program (Oncogrid) connecting primary care dental practitioners and frontline health workers (FHW) with oral cancer specialists for screening oral cancer.	The specialist reviewed the image and judged it as interpretable or not interpretable. The interpretable images were clinically stratified as nonneoplastic, potentially malignant, or malignant. Oncogrid Network of mobile phone. Sana platform (Computer & AI)	Oral Cancer Specialist Target screening: FHW (*n* = 4): 2000 patients, Opportunist screening Dentist: 1440 patients	Concordance with dentist:100 Positive predictive value:100 Concordance with FWH: Positive predictive value:45
Haron et al., 2017, Malaysia [37]	To examine the concordance in clinical diagnosis of OPMD and referral decisions between dentists and oral medicine specialist (OMS)	Mobile: 3 types of phones with different cameras Dentists (*n* = 3); Oral Medicine Specialists (OMS) (*n* = 2)	OPMD: 8 Non OPMD or Normal: 8	Concordance between OMS: Presence of lesion Sensitivity: 70 Specificity: 100 Category of lesion Sensitivity: 75 Specificity: 100 Referral decision Sensitivity: 81 Specificity: 100
Song et al., 2018, India [52]	To screen high-risk populations for oral cancer using smartphone-based intraoral dual-modality immunofluorescence imaging platform and classification of images obtained. In addition, to compare the performance of different CNN and transfer learning	Android Smartphone Luxeon LED to enable the autofluorescence imaging (AFI) and a white light imaging (WLI) CNN toolbox Luxeon UV: Transfer learning VGG-CNN-M VGG-CNN-S VGG-CNN-16	Training/validation Normal: 66/20 Suspicious ^2^: 64/20	Best performance with AFI-WLI: VGG-CNN-M Accuracy:86.9 Sensitivity:85.0 Specificity: 88.7
Uthoff et al., 2018, India [57]	To use the smartphone’s data transmission capabilities, and uploaded to a cloud server, where a remote specialist can access the images and make a diagnosis. Furthermore to classify images in suspicious and non suspicious	Android Smartphone Luxeon LED with AFI and a WLI CNN: VGG-CNN-M	Suspicious ^3^ vs. non-suspicious Normal: 33; Suspicious: 60 OSCC:6 CNN, normal:86/suspicious: 84	Remote specialist/CNN Sensitivity: 92.59/85.00 Specificity:86.67/88.75
Welikala, et al., United Kingdom. 2020 [61]	To detect and classify oral lesions in low risk and high risk, first in a phase of collection with bonding box annotations from clinicians and after classifying by deep learning.	Mobile Mouth Screening Anywhere (MeMoSA) Classification: ResNet-101: CNN Detection: Faster R-CNN	2155 images captured by MeMoSA App (normal, benign, OPMD, malignant) Clinician: 3–7 experts Training:1744 (Back propagation/stochastic gradient) Validation: 207 Testing: 204	Identification image that containing lesion (test): Precision:84.77 Recall: 89.51 F1Score: 87.07 Identification imaging that required referral (test): Precision:67.1 Recall: 93.8 F1Score: 78.3 Refer-low risk OPMD/cancer or high OPMD (test): Precision:26.4/14.7 Recall: 43.9/56.0 F1Score: 33/23.3

**Table 3:** Medical imaging technique. CNN: convolutional neural network. DL: deep learning. NN: neural network. OC: oral cancer. OSCC: oral squamous cell carcinoma. PNN: probabilistic neural network. ROI: region of interest. SVM: support vector machine. ^1^ Contrast, correlation, energy; homogeneity; entropy; sum of squares variance; inverse difference moment; sum average; sum variance; sum entropy; difference entropy. ^2^ Short-run emphasis; long-run emphasis; low gray-level run emphasis; high gray-level run emphasis; short-run low gray-level emphasis; short-run high gray-level emphasis; long-run low gray-level emphasis; long-run high gray-level emphasis; gray-level non-uniformity: run length non-uniformity; run percentage.

Authors, Year, Country, Reference	Aims of Study	Method. Classifier’s	Sample	Outcomes: Diagnostic Performance (%)
Bourass et al., 2015. Morocco [31]	To develop computer-aided diagnostics systems that aims at providing a classification of suspicious regions content-based image retrieval (CBIR).	SURF: Speed Up Robust Features Hierarchical-SVM vs. RGB-Histogram	Facial & Oral cancer database: 4160 images.	Hierarchical model SVM feedback, Precision: 82
Chan, et al., 2019, Taiwan [32]	To develop the texture map based on branch-collaborative network model to allow detection cancerous regions and marking the ROI	SMOTE texture-map-based branch Network Wavelet transformation Gabor filtering Fully Convolutional Network (FCN) Feature Pyramidal Network (FPN)	(Training/Validity)/Testing Cancer: 25/5 Normal: 45/5	Branch Network/Gabor filter (ROI) Sensitivity: 0.9687/0.9314 Specificity: 0.7129/0.9475
Fu, et al., 2020 China [35]	To develop a rapid, non-invasive and easy-to-use DL approach to identifying OSCC using photograms.	CNN	Training / Internal validation/external validation *n* = 1469 images from hospital *n* = 420 (images from journal) external validation (*n* = 666)	Algorithm/OC expert/medical student/non-medical student Accuracy: 92.3/92.4/87.0/77.2 Sensitivity: 91.0/91.7/83.1/76.6 Specificity: 93.5/93.1/90.7/77.8
Jeyaraj & Nadar, 2019. India [39]	To develop a DL algorithm for automated, computer-aided oral cancer detecting system by investigating patient hyperspectral images	Partitioned Deep CNN SVM Deep belief Network	OC vs. Benign Partitioned CNN vs. expert oncologist (*n* = 100 images) OC vs. Normal Partitioned CNN vs. expert oncologist (*n* = 500 images)	Accuracy: 91.4 Sensitivity: 94 Specificity: 91 Accuracy: 94.5 Sensitivity: 94 Specificity: 98
Jurczyszyn & Kozakiewicz, 2019. Poland [40]	To formulate a differential diagnosis for leukoplakia vs. lichen planus in the oral mucosa based on digital texture analysis in intraoral macrophotography	Neural Network Bayesian (PNN) Run/short length emphasis matrix Co-occurrence matrix	Oral leukoplakia: 21 Oral lichen planus: 21 Normal: 21	Sensitivity: 57 Specificity: 74 Sensitivity: 38 Specificity: 81 Sensitivity: 94 Specificity: 88
Jurczyszyn et al., 2020. Poland [41]	To propose an effective texture analysis algorithm for oral leukoplakia diagnosis	PNN Run length matrix (short/long) Co-occurrence matrix (entropy/difference entropy) Haar wavelet transformation (Energy 5.6)	Oral leukoplakia:35	Sensitivity: 100 Specificity: 97
Shamim et al., 2019 Saudi Arabia [50]	To apply and evaluate the efficacy of six model for identifying pre-cancerous tongue lesions directly using photographic images to diagnose	Deep CNN Transfer learning: AlexNet; GooLeNet; Vgg19; Inceptionv3; ResNet50; Squeeze	Training (160 images, 80%) Validation (40 images, 20%) Tongue diseases (Internet images) Physician with more than 15 years of clinical practice	Best (benign or precancerous: VGG19)/4 benign and 1 precancerous: ResNet) Accuracy: 98/97 Sensitivity:89 Specificity:97
Spyrodonos et al., 2015. Greece. [53]	To determine robust macro-morphological descriptors of the vermillion border from non-standardized digital photographs and to exploit a probabilistic model for solar cheilosis recognition	Relevance vector machine	Solar cheilosis: 75 Non-solar cheilosis:75	Sensitivity: 94.6 Specificity: 96
Thomas et al., 2017 India [56]	To distinguish between different groups of carcinoma of different areas of oral cavity by different selected features of Grey Level.	Backpropagation Artificial NN (to validate): Grey Level Co-occurrence Matrix (GLCM) ^1^ Grey Level Run-Length Matrix (GLRL) ^2^ Boxplot analysis	Oral cancer vs. normal Training: *n* = 12 Validation: 4 Sections of images:192	Accuracy Selected 11features: 97.9 All 61 features: 91.6 GLCM: 89.5 GLRL: 85.4

**Table 4:** Fluorescence imaging. ANN: artificial neural network. AUC: area under the curve. CI: clinical impression. CNN: convolutional neural networks. DTC: decision tree classifier. ED: Epithelial dysplasia. EH: epithelial hyperkeratosis. FlS: fluorescence spectroscopy. GLCM: gray-level co-occurrence matrices. KLCC: Karhunen–Loeve linear classifier. kNN: k-nearest-neighbors. LBP: local binary pattern. LDA: linear discriminant analysis. LDA: linear discriminant analysis. LR: logistic regression, NN: neural network. OML: oral mucosal lesion. OPMD: oral potentially malignant disorders. OSCC: oral squamous cell carcinoma. OSF: oral submucous fibrosis. PCA: principal components analysis. PLS-ANN: partial least squares and artificial neural network. RF: random forest. ROI: region of interest. RVM: relevance vector machine. RBF: radial basis function. SCC: squamous cell carcinoma. SVM: support vector machine. TB: toluidine blue. Yr: year.

Authors, Year, Country, Reference	Aim/no. of Predictor Variables	Method. Classifier	Sample	Outcomes: Diagnostic Performance (%)
Aubreville, et al., 2017 Germany [27]	To diagnose OSCC using deep learning on Confocal laser endomicroscopy (CLE) images	CLE Patch-extraction of images CNN RF-LBP; RF-GLCM	OSCC:12	AUC: Patch-extraction (validation) Accuracy: 88.3 Sensitivity: 86.6 Specificity: 90′
Awais et al., 2020. China [28]	To propose a method for the classification of OML and OPMDs based on a GLCM texture to take a biopsy	Velscope (ROI) GLCM LDA K-NN	*n* = 22 OML, OPMD	Accuracy: 83 Sensitivity: 85 Specificity: 84
de Veld et al., 2004. Netherlands [33]	To develop and compare algorithms for lesion classification and to examine the potential for detecting invisible tissue alterations.	Xe-lamp PCA ANN KLLC	Receiver-operator characteristic areas under the curve (ROC-AUCs) Patients = 155 Health: 96	PCA/ANN Accuracy: 96.5/98.3 Sensitivity: 92.9/96.5 Specificity: 100/100 Not distinguish benign vs. premalignant
Halicek et al., 2017. United States [36]	To compare automatic labeling of cancer and normal tissue applying hyperspectral images for using intraoperative cancer detection.	Xenon White light CNN SVM, k-NN, LR, DTC, LDA	37 OSCC	External validation training CNN Accuracy: 77 ± 21/96.8 Sensitivity: 77 ± 19/96.1 Specificity: 78 ± 16/96.4
Heintzelman et al., 2000. United States [38]	To determine optimal excitation–emission wavelength combinations to discriminate normal and precancerous/cancerous tissue, and estimate the performance of algorithms based on fluorescence.	Xenon (ƛ:472/350/499) PCA	OPMD/malignant: 11/17	Training //validation Sensitivity: FlS:90/CI: 100//FlS:100/CI: 100 Specificity: FlS:88/CI:83//FlS:98/CI:100
Majumder et al., 2005. India [44]	To compare evaluation of the diagnostic efficacy of the Relevance vector machine (RVM) and Support vector machine (SVM)	N_2_ laser (ƛ:337 nm: 375–700 nm) (Linear vs. RBF) RVM (Bayesian framework) vs. SVM (non-Bayesian)	OSCC: 16 Normal: 13	RVM (Linear/RBF)//SVM (Linear/RBF) Training Sensitivity (84/88)//(86/93) Specificity (93/95)//(91/96) Validation Sensitivity (86/91)//(86/93) Specificity (96/95)//(92/95)
van Staveren et al., 2000. Netherlands [58]	To apply an Artificial Network of autofluorescence spectra for classifying either homogeneous or non-homogeneous leukoplakia	Xe-lamp (ƛ:420nm) FullyNN	Leukoplakia: 22 Normal: 6	Abnormal vs. Normal//Homogeneous/Non-homogeneous/Normal Sensitivity: 86//73/64/100 Specificity: 100//82/94/86
Wang C et al., 2003. China [59]	To evaluate whether algorithm could discriminate premalignant (ED) and malignant (SCC) tissues from ‘‘benign’’	Xenon (ƛ:330nm) PLS-ANN (partial least squares and ANN	Normal:15, OSF: 30, EH: 26, ED: 13, SCC: 13 Sensitivity: 81 Specificity: 96;	Accuracy: Normal: 93 OSF: 97 EH: 62 ED & OSCC: 77
Wang X et al., 2020. China [60]	To develop a personalized computational model to predict cancer risk level of OPMDs and explore a potential web application in OPMD screening.	VEL scope TB staining Gini Index	*n* = 266; Follow-up 3 yr Training (3/5)/test (2/5) Model B (baseline) Model P (personalized) Expert	Training Model B/model P/experts Sensitivity:81 Specificity (Low grade): 92.31 Test Specificity (Low grade) Model B/Model P: 91.78 Experts: 86.30

**Table 5:** Exfoliative cytology. ^1^ Liquid-based exfoliative cytology (LBEC). ^2^ Scraped; ^3^ Brush. ^4^ Morphological features: solidity, roundness, circularity, convex area, major axis, minor axis, eccentricity, ratio. ^5^ Early disease: benign vs. more severe lesion. ^6^ Late disease: lesser severity vs. more severe lesions. ANN: artificial neural network. CF: peaks-closed forest. DI: DNA index. DIC: differential interference contrast. GLCM: gray-level co-occurrence Matrix.KNN: k-neural neighbor algorithm. LR: regulated logistic regression. LASSO: least absolute shrinkage and selection operator: OCRIP: oral cancer risk index. OLK: oral leukoplakia. OSCC: oral squamous cell carcinoma. PLR: penalized logistic regression. PMOL: potentially malignant oral lesion. POCOCT: point of care oral cytology. RF: random forest. SVM: support vector machine.

Authors, Year, Country, Reference	Aim	Method. Classifier	Sample	Outcomes: Diagnostic Performance (%)
Banerjee et al., 2016. India [29]	To classify cells and nucleus for diagnosis oral leukoplakia and cancer ^1^	SVM MATLAB	OLK:16 OSCC:23	Cell/nucleus Sensitivity: 100/89.9 Specificity: 100/100
Dey et al., 2016, India. [34]	To classify cellular abnormalities smokers vs. non-smokers and precancer ^2^	SVM Texture Features: GLCM (energy, homogeneity, correlation, contrast DIC) Morphological features ^4^ Gradient Vector flow Snake model k-means clustering	No smoking: 30 Smokers: 63 Pre-cancer: 26	Accuracy: 85.71 Sensitivity: 80.0 Specificity: 88.89
Liu et al., 2017. China. [43]	To improve the performance of the risk index of preexisting model for assessment for oral cancer risk in OLK ^3^	SVM Peals—Random Forest SVM full KNN, CF; RF OCRI	Training/Validation Normal: 18/102 OLK: 28/82 OSCC: 41/93 Follow up: 23 ± 20 months	Peaks RF Sensitivity: 100 Specificity: 100
McRae et al., 2020 (a). USA. [45]	To describe cytopathology tools, including machine learning algorithms, clinical algorithms, and test reports developed to assist pathologists and clinicians with PMOL evaluation and using a POCOCT platform. ^3^	SVM Lasso logistic regression Training: PCA Validation: *K*-NN	Benign OPMD Oral epithelial dysplasia OSCC	Accuracy: 99.3 Clinical algorithm. AUC Benign vs. mild dysplasia: 0.81 No lesion vs. malignancy: 0.97
McRae et al., 2020 (b). USA. [46]	To classify the spectrum of oral epithelial dysplasia and OSCC and to determine the utility of cytological signatures, including nuclear F-actin cell phenotypes. ^3^	Lasso logistic regression Training: PCA Validation: *K*-NN	OPMD OSCC health	AUC Early disease ^5^:0.82 Late disease ^6^:0.93
Sarkar et al., 2016. India. [49]	To develop a novel non invasive method for early cancer trend in habitual smoking ^1^	DIC Fluorescence microscopy Fuzzy trend (Mamdani): Risk of OPMD in smokers	OPMD smokers: 40 Non-smokers: 40 Control: 40	Positive correlation of smoking duration with early cancer risk: Correlation co-efficient: 0.86 Accuracy: 96 Sensitivity: 96 Specificity: 96
Sunny et al., 2019. India. [52]	To evaluate the efficacy of telecytology system in comparison with conventional cytology ^3^	Manual (telecytology 2 vs. ANN Inception V3, Implemented in Python RF, LR, Linear discriminant analysis, KNN	Training/Validation OPML: 3 OSCC: 3	SVM (best accuracy) Sensitivity: 88 malignant lesion: 93%, high grade OPML: 73% Specificity: 93
Wieslander et al., 2017, Suecia. [62]	To presents a pilot study on applying the PAP-based screening method for early detection of oral cancer and to compare 2 network architectures ^3^	Classifier: CNN Evaluation: ResNet VGG net	Herlev dataset Normal: 3 OSCC: 3	ResNet/VGGNet OSCC vs. normal Accuracy:78.3/80.6 vs. 82.3/80.8 Precision;72.4/75.0 vs. 82.4/82.4 Recall: 79.0/80.6 vs. 82.5/79.8 F score: 75.5/77.6 vs. 82.5/81.0

**Table 6:** Predictor (attributes) variable. FNN: fuzzy neural network. FR: fuzzy regression. KNN: K-nearest neighbors. MLP: multilayer perceptron. NN: neural network. OCC: oral cancer clinicians. SVM: support vector machine. NB: naïve Bayes. WEKA: Waikato Environment for Knowledge Analysis.

Authors, Year, Country, Reference	Aim	Method. Classifier	Sample (Prediction Factors)	Outcomes: Diagnostic Performance (%)
Karem et al., 2017, Malaysia. [42]	To guide oral cancer diagnosis using a real-world medical dataset with prediction model.	Training (PS-Merge) Fuzzy NN Fuzzy Regression Fuzzy Logic Logistic Regression	Oral Cancer: 171 (*n* = 8):	Best 7 factors Accuracy:78.95 Sensitivity: 100 Specificity: 58.62
Mohd et al., 2015, Malaysia. [47]	To predict more accurately the presence of oral cancer with reduced number of attributes.	SMOTE Features selection algorithm SVM Updatable Naïve Bayes Multilayer Perceptron K-Nearest Neighbors	Re-sample Oral cancer: 201 (*n* = 25):	Accuracy N° of features: NB/MLP/SVM/KNN 25: 91.9/94.2/93.3/86.1 14: 94.7/94.7/92.3/90.9
Rosma et al., 2010, Malaysia. [48]	To evaluate the ability of a fuzzy neural network (FNN) model and fuzzy regression (FR) model to predict the likelihood of an individual in developing OC based on knowledge of their risk habits and demographic profiles.	Prediction Model: FNN FR	Oral cancer: 84 Non-cancer: 87 (*n* = 5)	P value (Factors: 1 or 2/3 or 4) FR vs FNN: 1/1 FR vs. OCC: 1/0.043 FNN vs. OCC: 1/0.02
Sharma & Om, 2015, India. [51]	To design a data mining model using probabilistic and general regression neural network for early detection and prevention of oral malignancy.	Probabilistic NN/ General Regression NN	Oral cancer: 1025 (*n* = 12)	Benign vs malignant Accuracy: 99.0 Sensitivity: 99.3 Specificity: 98.0
Tetarbe et al., 2017, India. [55]	To analyze and classify data from an oral cancer dataset for accurate prognosis model from it.	WEKA Naïve Bayes J48Tree SMO algorithm REPTree Random Tree	Oral cancer:48 (*n* = 18)	Explorer/Experimenter Accuracy Naïve Bayes: 60.4/64.9 J48Tree: 75.0/77.6 SMO algorithm: 70.8/NA REPTree: 79.1/78.72 Random Tree: 72.8/68.3
